# Supplementary figures and images for: Entomo-Virological Surveillance and Genomic Insights into DENV-2 Genotype III Circulation in Rural Esmeraldas, Ecuador
Source: Pathogens. 2025 May 28;14(6):541. doi: 10.3390/pathogens14060541 (PMC12195760; doi:10.3390/pathogens14060541)

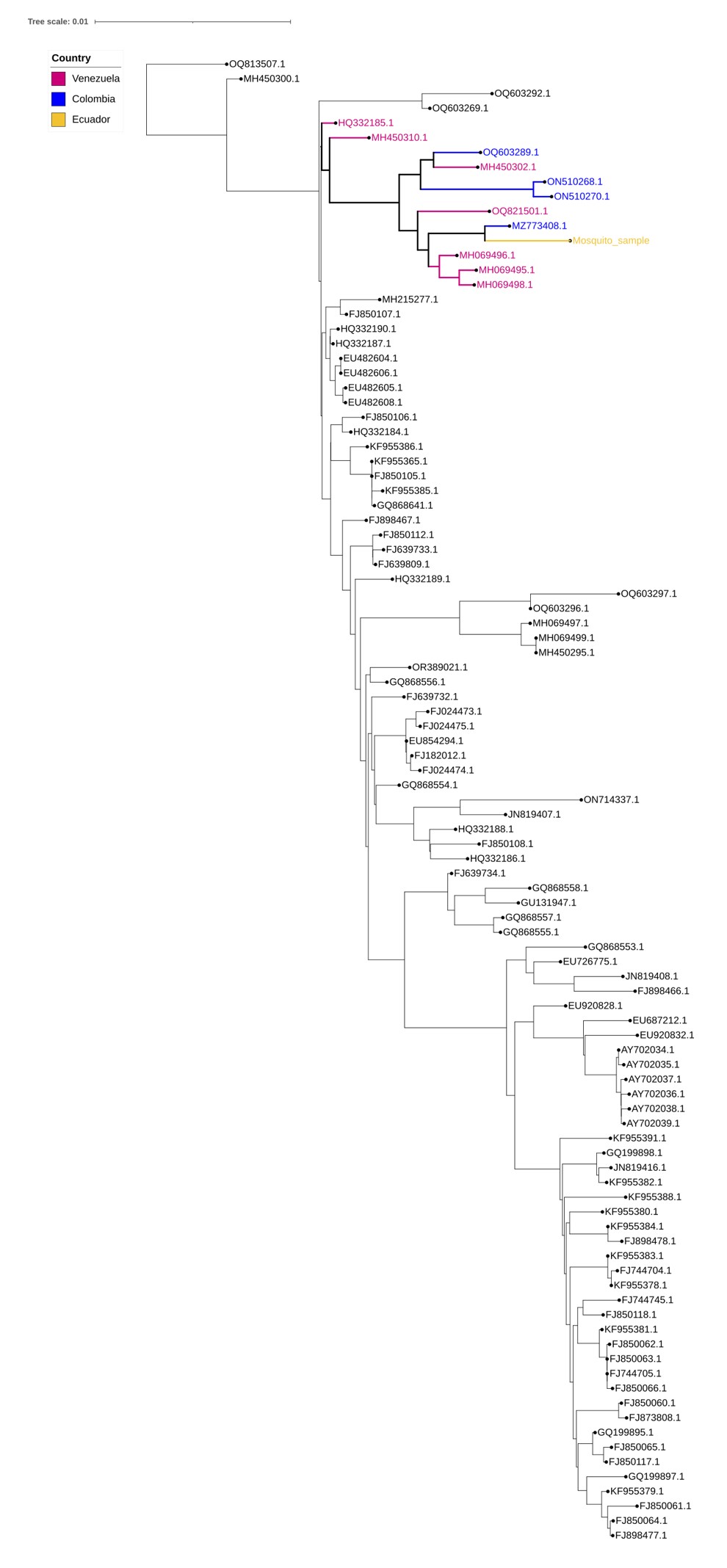

Supplement: Supplementary file 1 [file pathogens-14-00541-s001.zip › pathogens-3603511-supplementary/S1_Fig.jpg]
